# Association of *JAZF1* and *TSPAN8/*LGR5 variants in relation to type 2 diabetes mellitus in a Saudi population

**DOI:** 10.1186/s13098-015-0091-7

**Published:** 2015-10-24

**Authors:** Khalid Khalaf Alharbi, Imran Ali Khan, Rabbani Syed, Fawiziah Khalaf Alharbi, Abdul Khader Mohammed, Benjamin Vinodson, Nasser M. Al-Daghri

**Affiliations:** Department of Clinical Laboratory Sciences, College of Applied Medical Sciences, King Saud University, P.O. Box 10219, Riyadh, 11433 Kingdom of Saudi Arabia; Department of Biology Science, College of Science and Arts, Al-Qassim University, PO Box 1300, Buraidah, 51431 Kingdom of Saudi Arabia; Prince Mutaib Chair for Biomarkers of Osteoporosis, Biochemistry Department, College of Science, King Saud University, Riyadh, 11451 Saudi Arabia; Biomarkers Research Program, Department of Biochemistry, College of Science, King Saud University, Riyadh, 11451 Saudi Arabia

**Keywords:** T2DM, *JAZF1*, *TSPAN8/*LGR5, Saudi population

## Abstract

**Background:**

Type 2 diabetes mellitus (T2DM) is a chronic and multifactorial disease with a rapidly rising incidence in Saudi Arabia. Various genes including zinc finger protein 1 (*JAZF1*) and tetraspanin 8/leucine-rich repeat-containing G protein-coupled receptor (*TSPAN8/LGR5*) have been previously described to be associated with T2DM. This study investigated the association of *JAZF1* (rs864745) and *TSPAN8* (rs7961581) with T2DM in a Saudi population.

**Methods:**

Genomic DNA samples from 400 Saudi T2DM patients and 400 healthy controls were genotyped and analyzed using a polymerase chain reaction-restriction fragment length polymorphism method. The difference between the genotype frequencies were carried out with Chi-square test. Odds ratio, 95 % confidence intervals and p values were calculated using multinomial logistic regression. Dominant and recessive models were implemented to show the statistical significances. Analysis of variance was used to compare differences between genotypes for the various parameters.

**Results:**

Distribution frequencies of the AA, AG, and GG genotypes of *JAZF1* (rs864745) differed significantly among T2DM patients and healthy controls (*p* < 0.05). The AG and GG genotypes were independently and significantly associated with a T2DM risk after adjusting for factors such as age, sex, and body mass index [odds ratio (OR) 2.1 (95 % confidence interval (CI) 1.3–3.4); *p* = 0.002] and [OR 1.9 (95 % CI 1.2–3.1); *p* = 0.005], respectively. A genotype-based stratification of anthropometric and biochemical data revealed that the AG + GG genotype is associated with waist circumference (*p* = 0.04) and fasting blood glucose (*p* = 0.01) and high-density lipoprotein cholesterol levels (*p* = 0.02). None of the allele or genotype showed the significant association between the T2DM cases and control subjects in rs7961581 polymorphism in TSPAN8*/LGR5* gene.

**Conclusion:**

The rs864745 variant in *JAZF1* gene may act as genetic risk factors for the development of T2DM in a Saudi population.

## Background

Type 2 diabetes mellitus (T2DM) is a multifactorial and common chronic metabolic disease that is a major public health concern in many countries including the Arab population [1]. An estimated number of 285 million people are currently diabetic worldwide, and this is expected to increase up to 438 million by the year 2030. [2–4]. Worldwide, Saudi Arabia ranks seventh in T2DM prevalence and the incidence of this disease has doubled over the last two decades in the kingdom [1]. Several risk factors such as obesity, hypertension, undesirable lipid profiles, and increased fasting glucose levels as well as a parental history of diabetes contributes the predisposition for T2DM development [5]. Besides lifestyle factors and other environmental parameters which are partially responsible for the increased T2DM incidence, also genetic risk factors were shown to contribute [6]. The increased incidence of T2DM and the importance of early detection and management have prompted investigations to identify environmental and genetic factors that increase T2DM, risk and the related complications [7].

New insights into the genetic risk for T2DM include variants of the genes juxtaposed with another zinc finger protein 1 (*JAZF1*) and tetraspanin 8/leucine-rich repeat-containing G protein-coupled receptor (*TSPAN8/LGR5*) [7–9]. Numerous replication studies have indicated that *JAZF1* and *TSPAN8/*LGR5 affect various aspects of β-cell function [9–13].

The rs864745 variant in *JAZF1* and rs7961581 variant in *TSPAN8/LGR5* are significantly associated with insulin secretion and glucose-tolerance [14] and T2DM risk [9, 15]. However, there is limited information available on possible association of rs864745 (*JAZF1*) and rs7961581 *(TSPAN8/LGR5*) polymorphism with T2DM susceptibility in Saudi population. Therefore, in this case–control study, we tested whether the polymorphisms in *JAZF1* (rs864745) and *TSPAN8/*LGR5 (rs7961581) represents an additional risk factor for T2DM.

## Methods

### Patient selection

A total of 800 Saudi subjects (400 T2DM patients and 400 healthy controls) belonging to the same demographic area were enrolled in this study as described previously [16]. T2DM diagnosis was based on the criteria of World Health Organization (fasting glucose level ≥126 mg/dL or >7.0 mmol/L). A generalized questionnaire was administered to all consenting subjects and included demographics and past medical history. The subjects with other medical complications such as coronary artery disease, nephropathy, thyroid, and liver or end stage renal diseases were excluded from this study. Healthy volunteers (*n* = 400) with normal fasting blood glucose levels were selected from the general population based on age and gender. From the total recruited subjects 88.5 % of the T2DM and 52.5 % of the controls had a family history of T2DM. The study was approved by the local ethics committee [1, 5].

### Sample collection

Five milliliters (mL) of over-night fasting blood samples were obtained, 2 mL were collected in ethylenediamine tetra acetic acid (EDTA) tubes (used for molecular analysis) and the other 3 mL in serum separator tubes.

### Clinical and biochemical estimations

Clinical and anthropometric parameters, including blood pressure (mmHg), weight (kg), height (m), and hip (cm) and waist (cm) circumferences were measured using standard procedures [17]. Body mass index (BMI) was calculated as weight/height^2^ (kg/m^2^). Fasting blood glucose (FBG) and lipid profile [triglyceride (TG), total cholesterol (TC), and high-density lipoprotein cholesterol (HDL-C)] were measured using a chemistry auto-analyzer and commercially available kits (Konelab, Finland). Low-density lipoprotein cholesterol (LDL-C) concentrations were calculated using Friedwald’s formula. Insulin was quantified by a solid phase enzyme amplified sensitivity immunoassay (Medgenix INS-ELISA, Biosource, Belgium). Homeostasis model assessment for insulin resistance (HOMA-IR) was calculated as follows = insulin (μU/mL) × glucose (mmol/L)/22.5. Dyslipidemia (low levels of HDL-C) was defined as an HDL-C level of <1.03 mmol/L for men and <1.29 mmol/L for women [5, 16, 17].

### DNA extraction

Genomic DNA was isolated from whole blood using a standard DNA extraction kit (Norgen Biotek Corp., Canada). The quality and quantity of DNA were examined using Nanodrop and gel electrophoresis. DNA was stored at −80 °C.

### Analysis of polymorphisms

In this study, we have opted 2 genetic variants, such as *JAZF1* (rs864745) and *TSPAN8*/*LGR5* (rs7961581) for performing the molecular analysis based on previous literature studies with higher minor allele frequency (MAF) [9, 11–13]. Genotyping was carried out using the polymerase chain reaction-restriction fragment length polymorphism (PCR–RFLP) method. For rs864745 (A > G), primers 5′-GAGCCATATAAGTGATGCTCAAA-3′ (sense) and 5′-GGTTGTCAGGCTTTCCATGT-3′ (antisense) were used to amplify a 378-bp DNA fragment, whereas for rs7961581 (T > C), primers 5′-TGGCATCCAGCTTGTTATTG-3′ (sense) and 5′-TGAGAGCACTGTGTGTGTCACT-3′ (antisense) were used to amplify a 243-bp DNA fragment. PCRs were performed in a thermal cycler with initial denaturation at 95 °C for 5 min, followed by 35 cycles of denaturation at 95 °C for 30 s, annealing 58 °C for 30 s, and extension at 72 °C for 45 s. For rs864745, the PCR product (15 µL) was digested with 2U *SSP*I restriction endonuclease (New England Bio Labs, UK) for 16 h at 37 °C. The 378-bp (A allele) PCR product was cleaved into 338 and 40-bp fragments in the presence of a G allele (rare), but not in its absence. For rs7961581 polymorphisms, 15 µL of PCR product was digested with 4U *Hpy*16611 (New England Bio Labs) for 18 h at 37 °C, producing fragments of 210 and 33 bp in the case of a polymorphic variant (allele C) and T allele (243 bp) represents homozygous variant. All digested fragments were directly analyzed by polyacrylamide gel electrophoresis and visualized after silver staining [18].

### Statistical analysis

Statistical analyses were performed using SPSS version 19.0 for Windows (SPSS Inc., Chicago, IL, USA). All continuous variables were presented as mean ± standard deviation. An independent sample *T* test was used to compare the control and T2DM groups. Significant differences were defined as *p* < 0.05. Genotype frequency differences between patients and controls for each single nucleotide polymorphisms (SNPs) were tested using Chi square test. Odds ratios (ORs) and 95 % confidence intervals (95 % CIs) were calculated using multinomial (multiple) logistic regression with the wild type as the reference. Variables such as age, gender and BMI were included in the multinomial logistic model. Analysis of variance (ANOVA) was used to compare differences between genotypes for the various parameters. Chi-square test was used to identify departures from Hardy–Weinberg equilibrium.

## Results

Clinical and anthropometric data of subjects are shown in Table 1. Subjects with T2DM were significantly older and had higher BMI, waist and hip circumferences, systolic and diastolic blood pressure, and FBG, TG, and TC levels and lower HDL-C levels than control subjects.

**Table 1:** Epidemiologic, anthropometric and metabolic characterization of the individuals enrolled in the study

	T2DM (*n* = 400)	Controls (*n* = 400)	*p* value
Age (years)	53.6 ± 10.8	46.06 ± 7.7	<0.001
Sex (male/female)	232/168	211/189	0.13
Body mass index (kg/m^2^)	30.7 ± 6.3	29.2 ± 5.5	0.001
Waist (cm)	94.5 ± 22.2	91.6 ± 19.9	0.02
Hip (cm)	110.79 ± 18.2	101.46 ± 7.80	0.001
SBP (mmHg)	124.3 ± 11.2	114.9 ± 7.7	<0.001
DBP (mmHg)	78.4 ± 6.9	75.6 ± 6.0	<0.001
FBG (mmol/L)	12.9 ± 4.6	5.25 ± 0.59	<0.001
Triglycerides (mmol/L)	2.23 ± 1.2	1.61 ± 0.86	<0.001
Cholesterol (mmol/L)	5.6 ± 1.2	5.1 ± 0.98	<0.001
HDL-cholesterol (mmol/L)	0.83 ± 0.36	0.63 ± 0.22	<0.001
LDL-cholesterol (mmol/L)	3.8 ± 1.0	3.68 ± 0.85	0.18
Insulin (µU/mL)	16.2 ± 2.2	12.3 ± 1.7	0.006
HOMA-IR	7.12 ± 2.4	2.87 ± 1.7	<0.0001
Family history	354 (88.5 %)	210 (52.5 %)	<0.0001

*SBP* systolic blood pressure, *DBP* diastolic blood pressure, *FBG* fasting blood glucose

### Genotype distribution

Distribution of genotypes and allele frequencies of the rs864745 (*JAZF1*) polymorphism in patients and controls were in accordance with the Hardy–Weinberg equilibrium (p > 0.05), but not rs7961581 (*TSPAN8/*LGR5) (p < 0.01). The estimated powers of the study were 86 and 12.2 % (at desired significance level 0.05) for rs864745 and rs7961581, respectively.

Table 2 displays the genotype distribution for rs864745 and rs7961581 among the T2DM and control subjects. The distributions of the AA, AG, and GG genotypes (*p* < 0.005) and the AG + GG genotype (*p* < 0.0001) of rs864745 differed significantly between T2DM and control subjects. Likewise, the frequency AA genotype occurrence (rs864745) was significantly higher in the controls than the T2DM group (47.25 vs. 34.25 %), whereas the occurrence of the AG and GG genotypes was significantly higher in the T2DM group than controls (41.25 vs. 46.5 % for AG; 11.5 vs. 19.25 % for GG). No difference was observed between groups in the genotypic distribution of the rs7961581 variant.

**Table 2:** The genotype distribution of rs864745 (*JAZF1*) and rs796158 (*TSPAN8/LGR5*) polymorphisms in T2DM and control group

	Control n (%)	T2DM n (%)	Odds ratio^a^ (95 % CI)	p value	Odds ratio^b^ (95 % CI)	p value
*JAZF1* (rs864745)						
AA	189 (47.25)	137 (34.25)	Reference		Reference	
AG	165 (41.25)	186 (46.5)	1.5 (1.2, 2.1)	0.004	2.1 (1.3, 3.4)	0.002
GG	46 (11.5)	77 (19.25)	2.2 (1.4, 3.4)	0.0001	1.9 (1.2, 3.1)	0.005
AG + GG	211 (52.75)	263 (65.75)	1.7 (1.2, 2.1)	0.0001	2.0 (1.3, 3.2)	0.001
AA + AG	354 (89.5)	323 (80.75)	0.5 (0.3, 0.8)	0.002	0.8 (0.4, 1.5)	0.003
*TSPAN8* (rs7961581)						
CC	63 (15.8)	55 (13.8)	0.90 (0.59, 1.4)	0.63	1.0 (0.66, 1.6)	0.86
TC	138 (34.4)	153 (38.2)	1.1 (0.84, 1.4)	0.37	1.2 (0.82, 1.6)	0.41
TT	199 (49.8)	192 (48.0)	Reference		Reference	
CC + TC	201 (50.2)	208 (52.0)	1.07 (0.81, 1.4)	0.72	1.1 (0.82, 1.5)	0.47
TT + TC	337 (84.2)	345 (86.2)	1.1 (0.79, 1.73)	0.42	1.1 (0.81, 1.75)	0.38

^a^Crude odds ratio (95 % CI)

^b^Odds ratio (95 % CI) controlled for age, gender and BMI

We next determined the association of AG, GG, and AG + GG genotypes of rs864745 with T2DM risk, with AA as the (wild type) reference genotype (Table 2). Occurrence of the AG, GG, and AG + GG genotypes was associated with increased T2DM risk [ORs 1.5 (95 % CI 1.2–2.1, *p* = 0.004); 2.2 (95 % CI 1.4–3.4, *p* < 0.0001), and 1.7 (95 % CI 1.2–2.1, *p* < 0.0001), respectively]. No association was found for the rs7961581 variants (p > 0. 05). We next evaluated the association between *JAZF1* or *TSPAN8/*LGR5 variants and T2DM risk using multiple logistic regression analysis for independence after controlling for other risk factors such as age, gender, and BMI. A significant association was detected for the AG, GG, and AG + GG genotypes of rs864745 (ORs 2.1 (95 % CI 1.3–3.4, *p* = 0.002); 1.9 (95 % CI 1.2–3.1, *p* = 0.005), and 2.0 (95 % CI 1.3–3.2), *p* = 0.001, respectively). Conversely, no effect of rs7961581 on T2DM susceptibility was observed.

Table 3 shows the stratification of the genotype distribution for the rs864745 and rs7961581 variants based on gender. The AG and AG + GG variants of *JAZF1* (rs864745) were consistently associated with increased T2DM risk in both female [AG: OR 2.0 (95 % CI 1.2–3.9), *p* = 0.02); AG + GG: OR 1.9 (95 % CI 1.1–3.6), *p* = 0.03] and male subjects (AG: OR 1.8 (95 % CI 1.0–3.1), *p* = 0.04; AG + GG: OR 1.7 (95 % CI 1.0–2.9), *p* = 0.03). This effect remained significant only in males (*p* < 0.01) even after adjusting for age and BMI. Finally, the gender-based stratification of the genotype distribution for rs7961581 suggested that the association with T2DM is driven by male gender. The association remained significant even after adjusting for age and BMI (TC: OR 1.8 (95 % CI 1.1–2.9), *p* = 0.01; TC + CC: OR 1.7 (95 % CI 1.1–2.6), *p* = 0.01).

**Table 3:** The genotype distribution of rs864745 (*JAZF1*) and rs796158 (*TSPAN8/LGR5*) polymorphisms in T2DM and control group stratified based gender

	Males				Females			
	Odds Ratio (95 % CI)^a^	p value	Odds ratio (95 % CI)^b^	p value	Odds Ratio (95 % CI)^a^	p value	Odds ratio (95 % CI)^a^	p value
JAZF1 (rs864745)								
AA	Reference				Reference			
AG	2.0 (1.1, 3.9)	0.02	2.0 (1.0, 4.0)	0.04	1.8 (1.0, 3.1)	0.04	2.2 (1.1, 4.3)	0.01
GG	1.8 (0.96, 3.5)	0.06	1.7 (0.87, 3.5)	0.11	1.7 (0.98, 3.0)	0.05	2.2 (1.2, 4.2)	0.01
AG + GG	1.9 (1.1, 3.6)	0.03	1.9 (0.99, 3.6)	0.05	1.7 (1.0, 2.9)	0.03	2.2 (1.2, 4.1)	0.008
TSPAN8 (rs7961581)								
CC	0.64 (0.33, 1.2)	0.19	0.72 (0.36, 1.4)	0.35	1.1 (0.66, 1.9)	0.62	1.4 (0.76, 2.5)	0.27
TC	0.74 (0.47, 1.2)	0.21	0.69 (0.42, 1.1)	0.14	1.6 (1.1, 2.4)	0.02	1.8 (1.1, 2.9)	0.01
TT	Reference		Reference		Reference		Reference	
CC + TC	0.72 (0.47, 1.1)	0.12	0.70 (0.45, 1.1)	0.12	1.5 (1.0, 2.1)	0.04	1.7 (1.1, 2.6)	0.01

^a^Crude odds ratio (95 % CI);

^b^Odds ratio (95 % CI) controlled for age and BMI

### Association of *JAZF1* and *TSPAN8/LGR5* variants with metabolic parameters

 To study the effects of *JAZF1* and *TSPAN8/LGR5* polymorphisms on the different clinical parameters, we analyzed the distribution of these variables in relation to the various genotypes. Compared with the AA genotype, the AG + GG genotype subjects had significantly higher waist circumference (93.8 ± 20.4 vs. 89.8 ± 22.1 cm, *p* = 0.04), FBG (9.3 ± 5.1 vs. 8.0 ± 4.7 mmol/L, *p* = 0.01) and lower HDL-C concentration (0.72 ± 0.31 vs. 0.79 ± 0.34 mmol/L, *p* = 0.02; Table 4). No such associations were found for the rs7961581 polymorphism, for any of the variables examined Table 5.

**Table 4:** Anthropometric and metabolic parameters according to genotype of *JAZF1* and *TSPAN8/LGR5* polymorphisms

	*JAZF1* (rs864745)			*TSPAN8/LRG5* (rs7961581)		
	AG + GG	AA	p value	TC + CC	TT	p value
N	474	326		409	391	
Age (Years)	49.8 ± 10.1	49.8 ± 10.2	0.99	49.6 ± 9.7	49.9 ± 10.5	0.69
BMI (kg/m^2^)	29.9 ± 5.7	29.8 ± 5.9	0.90	29.8 ± 5.8	29.9 ± 5.8	0.81
Waist (cm)	93.8 ± 20.4	89.8 ± 22.1	0.04	92.5 ± 20.3	94.1 ± 21.1	0.28
Systolic BP (mmHg)	119.7 ± 10.8	119.5 ± 10.3	0.83	120.1 ± 10.9	119.2 ± 10.5	0.21
Diastolic BP (mmHg)	77.0 ± 6.7	76.8 ± 6.2	0.71	77.1 ± 6.7	76.9 ± 6.5	0.70
FBG (mmol/L)	9.3 ± 5.1	8.0 ± 4.7	0.01	9.1 ± 5.1	9.0 ± 5.0	0.76
TG (mmol/L)	1.8 ± 1.1	1.9 ± 1.1	0.84	1.8 ± 1.0	1.9 ± 1.1	0.30
TC (mmol/L)	5.3 ± 1.2	5.5 ± 1.0	0.18	5.4 ± 1.2	5.3 ± 1.1	0.73
HDL-C (mmol/L)	0.72 ± 0.31	0.79 ± 0.34	0.02	0.73 ± 0.31	0.72 ± 0.32	0.52
LDL-C (mmol/L)	3.7 ± 0.98	3.7 ± 0.81	0.61	3.7 ± 0.97	3.7 ± 0.94	0.52

Data represented by mean ± standard deviation

**Table 5:** Evaluation of risk allele frequencies in the earlier ethnic populations

Region/populations	Gene	Gene	References
Locus	*JAZF1*	*TSPAN8/LGR5*	Cheng et al. [15]
Risk allele	A	C	Cheng et al. [15]
SNPs	rs864745	rs7961581	Cheng et al. [15]
African Americans	72.8	22.4	Cheng et al. [15]
Native Hawaiian	73.2	28.9	Cheng et al. [15]
White	51.9	28.6	Cheng et al. [15]
Chinese	76.9	20.8	Zhou et al. [9]
Japanese	78.4	20.5	Omiri et al. [23]
Indians	67.5	35.2	Sanghera et al. [35]
Europeans	50.1	26.9	Zeggini et al. [9]
Latino	61.7	21.3	Cheng et al. [15]
Japanese Americans	77.8	20.3	Cheng et al. [15]

## Discussion

To the best of our knowledge, the association between the *JAZF1* (rs864745) or *TSPAN8/*LGR5 (rs7961581) polymorphisms and T2DM has not been reported in Arab populations, particularly in Saudi Arabia, despite an increased prevalence and incidence of T2DM. The present study is the first to examine the interaction between silent substitutions with no alteration in amino acids and provides preliminary information on the association between *JAZF1* or *TSPAN8/*LGR5 polymorphisms and T2DM risk. Additionally, the AG + GG genotype of rs864745 was significantly associated with higher waist circumference and FBG as well as lower serum HDL-C concentrations.

The *JAZF1* polymorphism (rs864745) is within intron 1 of this gene, which interacts specifically with the ligand-binding domain of TR4 (known to play roles in lipid metabolism and gluconeogenesis) and functions as a TR4-selective cofactor that may be involved in mediating transcriptional repression by TR4 [14, 19, 20]. However, differences in the distribution of risk alleles in various ethnic populations have been reported [9–14]. A number of studies have found an association between certain *JAZF1* (rs864745) variants and T2DM, whereas others have found no association [21]. A Framingham Heart Study in whites found that variants of *JAZF1* (rs864745) are associated with increased risk for T2DM [22], but a meta-analysis in a Japanese population identified a nominal association between *JAZF1* (rs864745) variants and T2DM [23]. Certain variants in the *JAZF1* locus are associated abnormal pancreatic β-cell function, which plays a role in the pathogenesis of T2DM and are significantly associated with impaired glucose regulation as well as diabetic nephropathy [14, 19, 20, 24]. Our results are similar to those of the above studies showing that *JAZF1* (rs864745) variants are significantly associated with the risk of T2DM [9, 11, 13]. A previous study in the same local Saudi population showed that other *JAZF1* variants (rs849134) are associated with increased BMI and T2DM risk [1, 25]. In contrast, a study in a Japanese population reported no significant association between *JAZF1* polymorphisms (rs864745) and T2DM [26]. McCarthy et al. [27] have reported that T2DM-associated variants, including those in *JAZF1,* in healthy populations have a predominant effect on insulin secretion, whereas a study in nondiabetic Finnish men failed to confirm such an association with *JAZF1* [28].

The SNP associated with *TSPAN8* (rs7961581) have been mapped to a region 110-kb upstream of this gene, which encodes a widely expressed cell surface glycoprotein known to form complexes with integrins. The binding of 6-integrin to laminin negatively affects pancreatic β-cell mass maintenance [20], and SNPs in *TSPAN8* may influence pancreatic β-cell function [14, 19, 20]. Using the results from oral glucose tolerance tests, several studies are described an association between certain *TSPAN8*/LGR5 variants (rs7961581) and insulin release, reflecting altered β-cell function in T2DM [9, 29, 30]. A significant association between *TSPAN8/LGR5* rs7961581 and T2DM was found in East Asians and European populations [9, 23]. By contrast, analysis of different *TSPAN8* variants (rs7961581) in the present study and rs4760790 from a previous study [31] in the same population failed to show such an association. The discrepancy may be explained by the ethnic and allele frequency differences in various population. The insufficient study power (12.2 %) could also be a probable reason for lacking the association between rs7961581 and T2DM in Saudi population.

We examined various clinical and anthropometric characteristics in the study subjects and correlated them with the various *JAZF1* and *TSPAN8*/LGR5 genotypes (i.e., AG + GG vs. AA and TC + CC vs. TT). Only the AG + GG versus AA genotype comparison revealed an association with waist circumference, FBG, and HDL-C (*p* < 0.05). T2DM is associated with an increase in total TG and a decrease in HDL-C concentrations. Several population-based studies have shown that visceral fat accumulation, high levels of TGs, and low levels of HDL-C predict the development of T2DM [28, 32]. Indeed, the results from our study suggest that subjects with T2DM have increased waist circumference and TG concentrations and decreased HDL-C levels. To derive further support for the role of *JAZF1* (rs864745) and *TSPAN8/*LGR5 (rs7961581) variants in T2DM development, we analyzed the distribution of confounding factors in relation to possible genotypes. We found the AG + GG genotypes of rs864745 to be significantly associated with increased waist circumference, elevated FBG levels, and reduced levels of serum HDL-C, suggesting the possible participation of rs864745 polymorphism in T2DM etiology. The analysis by Hotta et al. [32] revealed no association of either polymorphism with visceral fat accumulation [19]. There is evidence to indicate an association between *JAZF1* variants (rs864745) and IGR risk, 2-h glucose concentrations, or both [33]. Furthermore, An et al. [22] have reported that *JAZF1,* independently or interactively with other genes, is associated with increased T2DM risk and TG/HDL-C ratio change across age groups. A study in Finnish men showed that *JAZF1* variants (rs864745) were nominally associated with an increase in blood lipoprotein levels [28]. Results from another study show that *JAZF1* variants are also associated with arteriolosclerosis [34].

The present study has certain limitations that need to be taken into account. A major limitation lies in the unmatched design of controls and T2DM cases. Control subjects were younger and leaner than T2DM patients.

## Conclusion

To our knowledge, the current study is the first to determine the distribution and frequency of rs864745 (*JAZF1*) polymorphisms and their association with T2DM risk, increased waist circumference, and decreased HDL-C concentrations in a Saudi population. This finding, along with those of a previous report [1] emphasize that *JAZF1* is a genetic susceptibility locus for the development of T2DM in Saudi populations. Functional analysis are necessary to identify the molecular basis underlying these genetic predispositions and phenotype application may provide useful strategies for predicting T2DM risk in this population.

## Abbreviations

T2DM: type 2 diabetes mellitus
*JAZF1*: juxtaposed with another zinc finger protein 1
*TSPAN8*: tetraspanin 8/leucine-rich repeat-containing G protein-coupled receptor
EDTA: ethylenediaminetetraaceticacid
BMI: body mass index
PCR: polymerase chain reaction
RFLP: restriction fragment length polymorphism
